# Diffusivities and Atomic Mobilities in BCC Ti-Fe-Cr Alloys

**DOI:** 10.3390/ma17081927

**Published:** 2024-04-22

**Authors:** Yi Huang, Jingjing Nie, Weimin Bai, Songsong Hu, Xinming Wang, Ligang Zhang, Libin Liu

**Affiliations:** 1 School of Materials Science and Engineering, Xiangtan University, Xiangtan 411105, China; 202121551397@smail.xtu.edu.cn (Y.H.); wn072197@163.com (J.N.); songhu@xtu.edu.cn (S.H.); wangxm@xtu.edu.cn (X.W.); 2 Key Laboratory of Materials Design and Preparation Technology of Hunan Province, Xiangtan University, Xiangtan 411105, China; 3 School of Materials Science and Engineering, Central South University, Changsha 410083, China; pdc@csu.edu.cn

**Keywords:** bcc-Ti-Fe-Cr alloys, atomic mobility, diffusion coefficient, CALPHAD

## Abstract

In this research, the diffusion behaviors within the Ti-Fe-Cr ternary system were examined at the temperatures of 1273 K and 1373 K through the diffusion couple technique. This study led to the determination of both ternary inter-diffusion and impurity diffusion coefficients in the body-centered cubic (bcc) phase for the Ti-Fe-Cr alloy, utilizing the Whittle–Green and Hall methods. The statistics show that the average diffusion coefficients ${\tilde{D}}_{FeFe}^{Ti}$ and ${\tilde{D}}_{CrCr}^{Ti}$ measured at 1273 K were 1.34 × 10^−12^ and 3.66 × 10^−13^, respectively. At 1373 K, the average values of ${\tilde{D}}_{FeFe}^{Ti}$ and ${\tilde{D}}_{CrCr}^{Ti}$ were 4.89 × 10^−12^ and 1.43 × 10^−12^. By adopting the CALPHAD method, a self-consistent database for atomic mobility in the bcc phase of the Ti-Fe-Cr system was established. This database underwent refinement by comparing the newly acquired diffusion coefficients with data from the existing literature. Diffusion simulations for the diffusion couples were performed, drawing on the established database. The error between the simulated diffusion coefficient and the experimental measurement data is within 15%, and the simulated data of the component distance distribution and diffusion path are in good agreement with the experimental data. The simulations generated results that aligned well with the observed experimental diffusion characteristics, thereby affirming the reliability and accuracy of the database.

## 1. Introduction

Over the past few decades, the excellent performance of titanium and its alloys, such as their specific strength, relatively low density, superior resistance to corrosion, and favorable biocompatibility, have positioned them as materials of choice for both the aerospace and biomedical fields [1,2,3]. One of the main challenges of their broader application, compared to steel and aluminum, is the high cost of production [4,5,6]. This issue largely arises from the reliance on expensive β-type elements, like V, Mo, and Nb, instead of more affordable ones, such as Fe, Cr, and Mn. Opting for cost-effective β-stabilizers is a strategic method to decrease alloy expenses [7]. Notably, Cr has been identified as an efficient eutectoid element [8], and Fe is considered an economical option for achieving high-strength, low-density alloys. Consequently, several economical Ti-Fe-Cr alloys have been developed, such as Ti-4.3Fe-7.1Cr [9], Ti-4.2Fe-6.9Cr [10], and Ti-3.0Al-8.5Cr-2.0Fe [11].

The properties of titanium alloys are closely tied to the microstructures that develop from their processing and subsequent heat treatments. A considerable body of research has been focused on exploring the diffusion mechanisms of alloying elements within titanium alloys, particularly within binary and ternary compositions, to understand the underlying processes that govern microstructure formation in β-titanium alloys [12,13,14,15]. The enhancement of mechanical properties in sophisticated titanium alloy systems is fundamentally linked to managing complex microstructural formations through a variety of transformations, including solidification, precipitation, recrystallization, and grain growth, processes which are all influenced to varying degrees by diffusion activities. Diffusion is a migration phenomenon caused by the thermal motion of atoms in crystals, and it is an important method of material movement. At present, diffusion in continuous media can generally be described using Fick’s law, which is a normal diffusion; however, there are also some anomalous diffusion situations that cannot be described using Fick theory, known as non-Fickian diffusion [16,17,18,19]. Therefore, a comprehensive examination of how alloying elements diffuse within multi-component titanium-based alloys is essential. Despite the extensive studies into diffusion within Ti-Fe [12,20,21,22], Ti-Cr [12,13,23,24,25], and Fe-Cr [26,27] alloy systems, there remains a notable gap in the literature concerning the diffusion behaviors and thermodynamic evaluations specific to the Ti-Fe-Cr system, highlighting a crucial area for further investigation.

Acquiring a thorough comprehension of the thermodynamic and kinetic properties of titanium alloys is critical for elucidating how temperature, duration, and alloy composition influence the microstructural evolution during heat treatment processes [28]. The CALPHAD methodology, which has seen substantial refinement, now reliably forecasts the thermodynamic and kinetic particulars for alloy systems incorporating an extensive range of elements, which are pivotal for their industrial relevance [29,30]. By integrating this approach, it is possible to assess and evaluate diffusion coefficients derived from the laboratory data, culminating in the establishment of an atomic mobility database. This database can offer exhaustive diffusion metrics across an entire gamut of compositions and temperatures. Through the application of thermodynamic equations and kinetic databases, facilitated by the CALPHAD model, the software DICTRA (Educational 2023b) [31,32] enables the meticulous prediction of diffusion processes in diffusion pairs, the homogenization of alloys, the kinetics of precipitation, and the progression of microstructural transformations [28,30,33]. This framework underpins precise simulations and predictions essential for optimizing the performance and application of titanium alloys in various industrial sectors. The study of alloy diffusion behavior based on the CALPHAD model has been successfully applied to a variety of alloy systems, such as steel, nickel-based alloys, aluminum alloys, and titanium alloys, and has shown important applications in alloy design and industrial production [29,30,31,32]. Companies such as Thermo_Calc [34] and CompuTherm [35] have established commercial atomic dynamics databases based on the CALPHAD model for a variety of alloy systems with a range of application paradigms. Based on the CALPHAD technique, the authors of the current study have studied the diffusion behavior and atomic mobility of several titanium alloy systems, such as Ti-Mo-Ta [36], Ti-Nb-Zr [37], Ti-Nb-Sn [38], etc., and established a database of atomic mobility of titanium alloys, which was used to simulate the α/β phase transition behavior in titanium alloys [39].

This study was carried out as follows: (i) A total of 11 pairs of Ti-Fe-Cr system bcc single-phase diffusion couples were prepared and annealed at 1273 K and 1373 K for a period of time. Electron probe micro-analysis was used to measure the concentration distribution curve of the diffusion interfaces to study the diffusion behavior of the elements in alloys. (ii) The Whittle–Green method was used to calculate the diffusion coefficient and to analyze the influence of alloy elements on the diffusion coefficient. (iii) The migration rate parameter database was optimized based on the CALPHAD method, and the diffusion process was simulated in DICTRA software. We compared the simulation results with the experimental results to verify the accuracy of the data library.

## 2. Experimental Procedure

**Figure 1:**
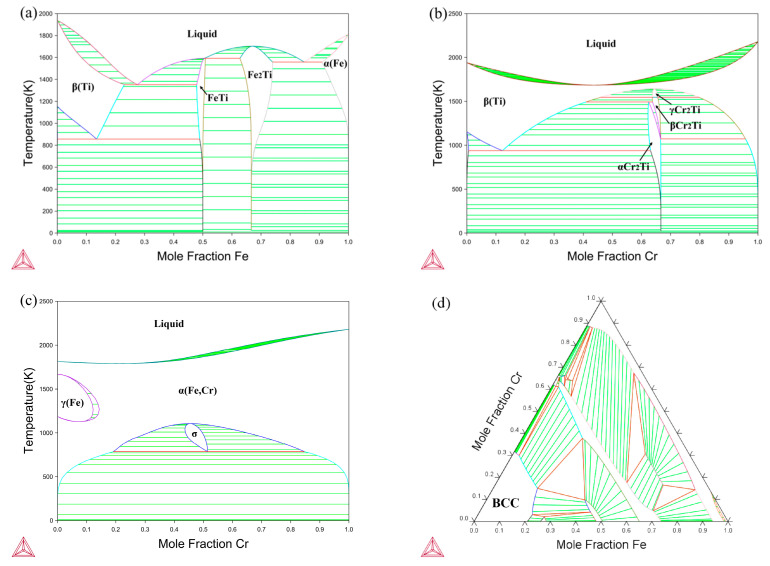
Phase diagrams of (**a**) Ti-Fe [40], (**b**) Ti-Cr [41], and (**c**) Fe-Cr [42] binary systems, and (**d**) isothermal sections of Ti-Fe-Cr [43] system at 1273 K.

The design of the alloy compositions was guided by the binary phase diagrams of the Ti-Fe, Ti-Cr, and Fe-Cr systems [40,41,42], along with the isothermal sections of the Ti-Fe-Cr [43] ternary system at 1273 K, as depicted in Figure 1. The chosen compositions reside within the bcc solid solution region of the Ti-Fe-Cr system at temperatures of 1273 K and 1373 K, as illustrated in Table 1. Alloys comprising solely Ti, binary mixtures of Ti-Fe, Ti-Cr, and the ternary Ti-Fe-Cr, were fabricated using arc melting in an argon environment. The melting stock included granules of Ti, Fe, and Cr, each with a purity of 99.99 wt%. The materials used in this experiment were provided by Shanghai Chunming Metal Materials Co., Ltd. in Shanghai, China. To achieve homogeneity of the compositions across the alloys, the melting procedures of all alloys were carried out six times. Following this, the ingots were subjected to vacuum annealing at 1473 K for 30 h before being quenched in ice water. This specific regimen led to the formation of alloys with a grain size averaging several millimeters, thereby significantly reducing the role of grain boundary diffusion.

The annealed ingots were shaped into rectangular blocks measuring 10 × 10 × 6 mm^3^ using WEDM. Following this process, one face of each block was polished until it achieved a mirror finish. The blocks were assembled as diffusion couples using steel clamps under vacuum at 1173 K for 2.5 h. To mitigate direct contact and reduce the risk of contamination, separators made of tantalum foil were strategically placed between the diffusion couples and the clamps. The assemblies were then securely sealed within quartz tubes, which were subsequently filled with argon of high purity. The encapsulated diffusion couples underwent annealing at temperatures of 1273 K for 44 h and 1373 K for 22 h. Then, they were quickly cooled by being submerged in ice water.

Following the annealing process, the diffusion couples were precisely divided along the diffusion path with the assistance of WEDM. This step was succeeded by their preparation through a series of standard metallography techniques, which included mounting, grinding, and polishing to achieve a suitable surface for examination. To further dissect the compositional gradients within these couples, electron probe microanalysis (EPMA, using the JXA-8230 model by JEOL, Tokyo, Japan) was employed. This analysis was conducted under specific conditions: a 15 kV operating voltage, a 20 nA beam current, and a 40-degree take-off angle, ensuring the detailed and accurate measurement of the composition–distance profiles.

## 3. Methodology

### 3.1. Extraction of Inter-Diffusion Coefficients

In the context of one-dimensional diffusion, the diffusion coefficients within multicomponent metallic solid solutions from the composition–distance profiles were calculated using the Matano–Kirkaldy method [44]. Kirkaldy [45] introduced the Boltzmann parameter $\lambda =z/\sqrt{t}$, where z represents the spatial position and t denotes the time, initially applying this parameter at the boundary condition when t=0, ${\left.c_{i}\right|}_{x<0}=c_{iL}$, ${\left.c_{i}\right|}_{x>0}=c_{iR}$:

(1) $$\begin{array}{c} {\tilde{J}}_{i}=\frac{1}{2t}\int_{c_{iL}}^{c_{i}}\left(z-z_{M}\right)dc_{i}=-\overset{2}{\underset{j=1}{\sum }}{\tilde{D}}_{ij}^{k}\frac{\partial c_{j}}{\partial z} \end{array}$$

The diffusion flux of an element i, symbolized by ${\tilde{J}}_{i}$, correlates with its concentration, $c_{i}$, where $c_{iL}$ stands for the element’s concentration at the diffusion couple’s left boundary. The parameter z reflects the distance from this boundary, $z_{M}$ denotes the position of the Matano plane, and t represents the elapsed time of diffusion. The symbol ${\tilde{D}}_{ij}^{k}$ refers to the diffusion coefficient of element i in medium k, influenced by the concentration gradient of element j. Within a ternary alloy system, it is essential to identify four independent diffusion coefficients: ${\tilde{D}}_{11}^{3}$, ${\tilde{D}}_{22}^{3}$, ${\tilde{D}}_{12}^{3},$ and ${\tilde{D}}_{21}^{3}$. To accurately determine these coefficients, employing two diffusion couples that converge at a singular intersection point is crucial.

Identifying the Matano plane, which is established as the zero-distance point, is the initial step in this method, yet this task is known for being both time-intensive and prone to inaccuracies. To address these challenges, Whittle and Green [46] proposed the use of a normalized concentration variable:

(2) $$Y_{i}=\frac{\left(c_{i}-c_{iL}\right)}{\left(c_{iR}-c_{iL}\right)}$$

In the study of diffusion processes, the concentration levels of species at the diffusion couple’s boundaries, denoted as $c_{iL}$ and $c_{iR}$ for the left and right ends, respectively, play a critical role. To refine the analysis of inter-diffusion fluxes, represented by ${\tilde{J}}_{i}$, a novel approach involves the utilization of a newly introduced variable, $Y_{i}$. This methodology facilitates the recalibration of the fluxes, enhancing the precision of the diffusion analysis.

(3) $${\tilde{J}}_{i}=\frac{c_{iR}-c_{iL}}{2t}\left[\left(1-Y_{i}^{*}\right)\int_{-\infty }^{z^{*}}Y_{i}dz+Y_{i}^{*}\int_{z^{*}}^{+\infty }\left(1-Y_{i}\right)dz\right]$$

where $Y_{i}^{*}$ is the $Y_{i}$ value at specific position $z^{*}$. Combining Equations (1) and (3), we can obtain in the Ti-Fe-Cr system:

(4a) $$\frac{1}{2t}{\left(\frac{dz}{dY_{Fe}}\right)}_{z^{*}}\left[\left(1-Y_{Fe}^{*}\right)\int_{-\infty }^{z^{*}}Y_{Fe}dz+Y_{Fe}^{*}\int_{z^{*}}^{+\infty }\left(1-Y_{Fe}\right)dz\right]={\tilde{D}}_{FeFe}^{Ti}+{\tilde{D}}_{FeCr}^{Ti}{\left.\frac{dc_{Cr}}{dc_{Fe}}\right|}_{z^{*}}$$

(4b) $$\frac{1}{2t}{\left(\frac{dz}{dY_{Cr}}\right)}_{z^{*}}\left[\left(1-Y_{Cr}^{*}\right)\int_{-\infty }^{z^{*}}Y_{Cr}dz+Y_{Cr}^{*}\int_{z^{*}}^{+\infty }\left(1-Y_{Cr}\right)dz\right]={\tilde{D}}_{CrCr}^{Ti}+{\tilde{D}}_{CrFe}^{Ti}{\left.\frac{dc_{Fe}}{dc_{Cr}}\right|}_{z^{*}}$$

The specific inter-diffusion coefficients at the point where the diffusion paths of two elements cross—namely ${\tilde{\;D}}_{FeFe}^{Ti}$, ${\tilde{\;D}}_{CrCr}^{Ti}$, ${\tilde{\;D}}_{FeCr}^{Ti},$ and ${\tilde{\;D}}_{CrFe}^{Ti}$—are determined by resolving Equation (4a,b). Given the scarcity of dependable information regarding how the composition influences the molar volume within Ti-Fe-Cr alloys, this research proceeded under the premise of a uniform molar volume across the board. The expectation is that the potential deviations stemming from this premise are sufficiently minor, thereby residing within the acceptable precision range for diffusion coefficients obtained via the Whittle–Green method.

### 3.2. Extraction of Impurity Diffusion Coefficients

Utilizing the Hall method [47], this study computed the impurity diffusion coefficients for chromium in the titanium–iron alloy and iron in the titanium–chromium alloy based on the final compositions of the diffusion pairs. This approach is notably effective for calculating inter-diffusion coefficients at the extremities of composition–distance profiles, where the Matano–Kirkaldy method encounters limitations due to the slope of the curve approaching zero, rendering the coefficients infinite. Initially, the composition profiles underwent a transformation to achieve a near-linear relation, depicted as μ=hλ+k. This step involved incorporating $\mu =erf^{-1}\left(2Y-1\right)$ and the Boltzmann parameter $\lambda =z/\sqrt{t}$. Following this, the analysis proceeded to extract parameters $h_{1}$, $k_{1}$ for the left and $h_{2}$, $k_{2}$ for the right sides of the diffusion pairs through linear fitting. Conclusively, this study utilized specific equations to accurately calculate the inter-diffusion coefficients at the endpoints of the diffusion couples.

(5a) $$\tilde{D}\left(x\prime \right)=\frac{1}{4{h_{1}}^{2}}\left[1+\frac{2k_{1}}{\sqrt{\pi }}\exp\left(\mu^{2}\right)\times Y\left(x\right)\right]$$

(5b) $$\tilde{D}\left(x\prime \right)=\frac{1}{4{h_{2}}^{2}}\left[1-\frac{2k_{2}}{\sqrt{\pi }}\exp\left(\mu^{2}\right)\times \left[1-Y\left(x\right)\right]\right]$$

$\tilde{D}\left(x\prime \right)$ specifically refers to the inter-diffusion coefficient at the left or right end of the profiles obtained when $Y\left(x\right)$ tends toward 0 or 1. If the composition of an element is 0, the impurity diffusion coefficient of that element is as follows:

(6) $$\underset{x\left(Zr\right)\to 0}{\lim}{\tilde{D}}_{ZrZr}^{Ti}=D_{Zr\left(\mathrm{Ti}-\mathrm{Sn}\right)}^{*}$$

(7) $$\underset{x\left(Sn\right)\to 0}{\lim}{\tilde{D}}_{SnSn}^{Ti}=D_{Sn\left(\mathrm{Ti}-\mathrm{Zr}\right)}^{*}$$

### 3.3. Atomic Mobility and Diffusion Coefficient

In the context of multi-component substitutional solutions, the diffusion flux experienced by any given species is directly affected by the thermodynamic forces at play. These forces manifest as gradients in chemical potential across the solution. Employing a synthesis of Fick’s first law with Onsager’s relational expressions enables the quantification of species-specific diffusion flux, symbolized by $J_{i}$. This approach elucidates the intricate relationship between thermodynamic gradients and the resultant diffusion behaviors.

(8) $$J_{i}=-\overset{n}{\underset{j=1}{\sum }}L_{ij}\left(\frac{\partial \mu_{j}}{\partial z}\right)=-\overset{n}{\underset{j=1}{\sum }}L_{ij}\frac{\partial \mu_{j}}{\partial c_{j}}\frac{\partial c_{j}}{\partial z}$$

By incorporating the inter-diffusion coefficient $D_{ij}^{n},$ it becomes feasible to articulate the diffusion flux, $J_{i}$, leveraging the phenomenological coefficient $L_{ij}$ alongside the chemical potential of species j, $\mu_{j},$ and the parameter z, representing distance.

(9) $$J_{i}=-\overset{n}{\underset{j=1}{\sum }}D_{ij}^{n}\frac{\partial c_{k}}{\partial z}$$

In scenarios where diffusion primarily occurs through mono-vacancy exchange mechanisms, the Einstein relation serves to clarify the correlation between the species-specific tracer diffusion coefficient, denoted as $D_{i}^{*},$ and the corresponding atomic mobility, represented by $M_{i}$. This pivotal relation sheds light on the influence of atomic mobility on the diffusion coefficient within contexts where vacancy exchange is the key mode of atom movement.

Within the context where mono-vacancy exchange predominates as the chief diffusion process, the Einstein relation provides a clarifying linkage between the tracer diffusion coefficient, $D_{i}^{*}$ for a given species i, and its atomic mobility, $M_{i}$. This relation demystifies how the mobility of atoms influences the diffusion coefficient in materials where vacancy-mediated diffusion is the primary mechanism.

(10) $$D_{i}^{*}=RTM_{i}$$

In a substitutional solution phase with a constant volume, the interdiffusion coefficient linked to atomic mobility is articulated through the following formula, where R represents the gas constant, and T the temperature in kelvins:

(11) $$D_{ij}^{n}=\overset{n}{\underset{k}{\sum }}\left(\delta_{ki}-x_{i}\right)x_{k}M_{k}\left(\frac{\partial \mu_{k}}{\partial x_{j}}-\frac{\partial \mu_{k}}{\partial x_{n}}\right)$$

The Kronecker delta $\delta_{ki}$ equals 1 when i = k, and is 0 otherwise; $x_{i}$, $x_{j}$, $x_{k},$ and $x_{n}$ denote the mole fractions of species i, j, k, and n, respectively; $M_{k}$ represents the atomic mobility of species k. Initially introduced by Andersson and Ågren [48] and further elaborated by Jönsson [49], the formula for the atomic mobility $M_{i}$ of species i in relation to temperature within disordered solid solutions is as follows:

(12) Mi=Mi0exp−QiRT1RT×Ω   mg=expRTlnMi0−QiRT1RT

The frequency factor for species i is denoted by $M_{i}^{0}$, and the activation energy is represented by $Q_{i}$. The term Ω   mg, indicative of the ferromagnetic contribution, is assumed to be 1, reflecting the absence of ferromagnetic transformation in the Ti-Fe-Cr alloys. Given that $∆G_{i}^{\varphi }=RT\ln M_{i}^{0}-Q_{i}$, Equation (12) can be reformulated as:

(13) $$M_{i}=\exp\left(\frac{∆G_{i}^{\varphi }}{RT}\right)\frac{1}{RT}$$

Using the CALPHAD approach, the parameter $∆G_{i}^{\varphi }$ is acknowledged to vary with the composition, and it is formulated using the Redlich–Kister polynomial [50] as follows:

(14) ∆Giϕ=∑pxpΦip+∑p∑q>pxpxq∑r=0,1,2,⋯Φ   rip,qxp−xqr       +∑p∑q>p∑v>qxpxqxv∑s=p,q,vΦ   sip,q,vxs+1−xp−xq−xv3

For species designated as p, q, and v, the corresponding mole fractions are labeled as $x_{p}$, $x_{q},$ and $x_{v}$. Furthermore, the symbol $\Phi_{i}^{p}$ denotes the mobility of species i when it is in its pure form, labeled as p. The parameters that describe interactions in mixtures with two and three components are respectively notated as Φ   rip,q for binary interactions and Φ   sip,q,v for ternary interactions. It is pertinent to note that within the framework of binary systems, the equation labeled as Equation (14) disregards the ternary interaction term, treating its value effectively as zero.

## 4. Results and Discussion

### 4.1. Composition–Distance Profiles and Diffusion Paths

**Figure 2:**
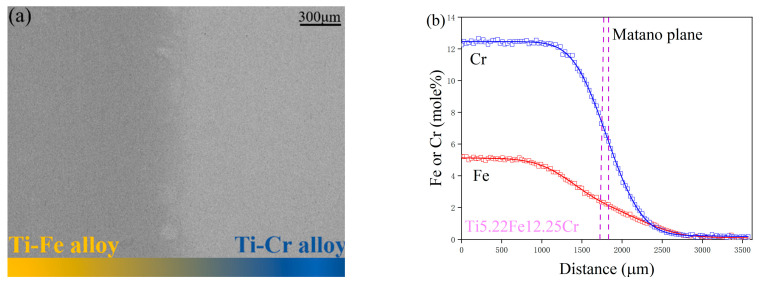
Depictions of (**a**) the diffusion zone through a SEM backscattered electron image and (**b**) the composition–distance profiles of the B1 diffusion couple after undergoing 44 h of diffusion at 1273 K.

Figure 2a showcases the microstructural view of the A1 diffusion couple, captured via backscattered electron microscopy, after a 44 h thermal treatment at 1273 K. Figure 2b illustrates the composition–distance profiles for the same couple, obtained through EPMA analysis. All diffusion couples within the Ti-Fe-Cr system are observed in a consistent bcc phase region, with the A1 microstructure serving as a prime example. The EPMA examination along a predetermined direction yielded accurate composition profiles, revealing that iron’s penetration depth significantly exceeded that of chromium, approximately reaching 2900 µm. This finding highlights iron’s substantially greater diffusion velocity compared to chromium in the β-phase Ti-Fe-Cr alloys.

Due to the discreteness of the experimental data points measured using EPMA, errors introduced in the fitting or smoothing process should be avoided. The composition–distance profiles obtained by EPMA are expressed analytically via using error function expansion (ERFEX) [37,51]:

(15) $$x\left(z\right)=\sum_{i}\left[a_{i}\mathrm{erf}\left(b_{i}z+c_{i}\right)+d_{i}\right]$$

**Figure 3:**
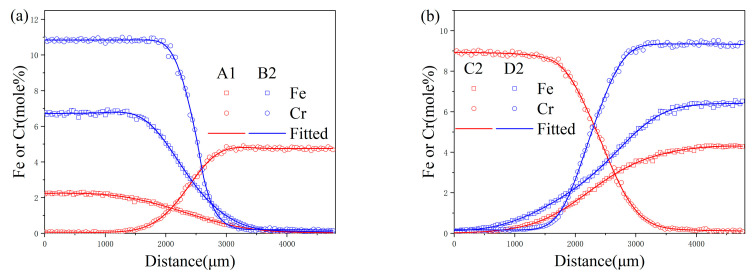
ERFEX depiction of diffusion profiles for (**a**) the A1-B2 diffusion couple after 44 h at 1273 K and (**b**) the C2-D2 diffusion couple following 22 h at 1373 K.

At the specified distance labeled z, the mixture’s composition is characterized by the parameters $a_{i}$, $b_{i}$, $c_{i},$ and $d_{i}$ each serving as variables for adjustment. Figure 3 provides a visualization of the composition versus distance profiles for diffusion couples, specifically for A1 and B2, which were maintained at a temperature of 1273 K for a duration of 44 h, and for C2 and D2, subjected to 1373 K for 22 h. These profiles are described utilizing the ERFEX model. In the execution of this study’s methodology, the selection of *i* = 4 for the ERFEX model was guided by the detailed examination of the diffusion couples’ composition–distance profiles. The employment of the error function in the analysis of diffusion processes offers a framework that is not only methodical but also imbues the findings with a more profound understanding of the physical phenomena being studied.

**Figure 4:**
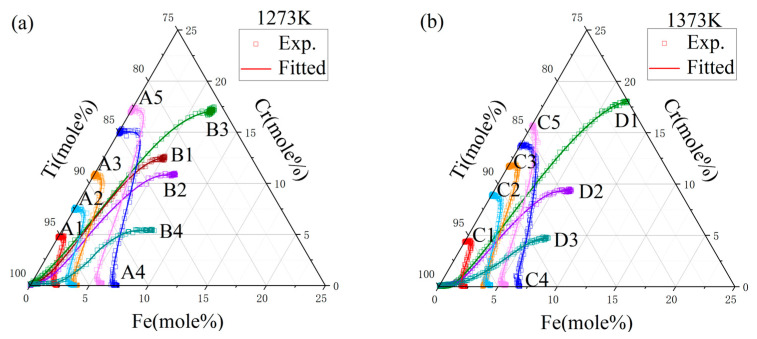
Diffusion paths of diffusion couples annealed at (**a**) 1273 K for 44 h and (**b**) 1373 K for 22 h.

In the study presented in Figure 4, diffusion paths for various couples were meticulously charted following their exposure to annealing at two distinct temperatures: 1273 K and 1373 K. The resulting diffusion trajectories prominently exhibited an S-shaped curve, indicative of the differential diffusion rates exhibited by Fe and Cr. Close examination of the graphical data revealed a total of 20 intersection points within the diffusion couples annealed at 1273 K, and 15 points at 1373 K. Employing the Whittle–Green method, the derivation of the ternary interdiffusion coefficients at the precise compositions coincide with these intersection points. In addition, the Hall method was used to calculate the impurity diffusion coefficients of chromium in titanium iron alloy and iron in titanium chromium alloy at the end of the A1–A5 and C1–C5 diffusion couples.

### 4.2. Diffusion Coefficients

Table 2, Table 3 and Table 4 detail the ternary diffusion coefficients in bcc Ti-Fe-Cr alloys, calculated at 1273 K and 1373 K using the Whittle–Green method. The standard deviations were determined from four independent calculations upon two independent measurements. This study further confirms that these coefficients, as derived through the Whittle–Green methodology, are in complete accord with the thermodynamic constraints outlined in reference [45]. These constraints are established as follows:

(16a) $${\tilde{D}}_{11}^{3}+{\tilde{D}}_{22}^{3}>0$$

(16b) $${\tilde{D}}_{11}^{3}\times {\tilde{D}}_{22}^{3}-{\tilde{D}}_{12}^{3}\times {\tilde{D}}_{21}^{3}\geq 0$$

(16c) $${\left({\tilde{D}}_{11}^{3}-{\tilde{D}}_{22}^{3}\right)}^{2}+4{\tilde{D}}_{12}^{3}\times {\tilde{D}}_{21}^{3}\geq 0$$

In the datasets presented in Table 2 and Table 3, the investigation clearly highlights that the predominant interdiffusion coefficients, specifically ${\tilde{D}}_{FeFe}^{Ti}$ and ${\tilde{D}}_{CrCr}^{Ti}$, significantly exceed the values of their cross-interdiffusion counterparts, ${\tilde{D}}_{FeCr}^{Ti}$ and ${\tilde{D}}_{CrFe}^{Ti}$. Notably, the primary coefficients are observed to be larger by one to two orders of magnitude when compared to the cross coefficients. This pattern underscores the dominant role of the intrinsic concentration gradients of Fe and Cr in steering their diffusion behaviors, with minimal influence from the concentration gradients of the other element. Furthermore, the derivations of the cross-interdiffusion coefficients, as facilitated by the Whittle–Green method, encounter substantial variability and oscillations. This outcome manifests a reduced level of precision and highlights the intrinsic variability associated with these calculations.

In Table 2, the value of the main interdiffusion coefficient ${\tilde{D}}_{FeFe}^{Ti}$ is between 0.79 × $10^{-12\;}m^{2}/s$ and 2.33 × $10^{-12\;}m^{2}/s$. In Table 3, the value of ${\tilde{D}}_{FeFe}^{Ti}$ is between 2.71 × $\;10^{-12}{\;m}^{2}/s$ and 6.33 × $10^{-12\;}m^{2}/s$. The average values of ${\tilde{D}}_{FeFe}^{Ti}$ at 1273 K and 1373 K are 1.34 × $10^{-12\;}m^{2}/s$ and 4.89 × $10^{-12}{\;m}^{2}/s$, respectively. The average values and ranges of ${\tilde{D}}_{FeFe}^{Ti}$, ${\tilde{D}}_{CrCr}^{Ti}$, $D_{Fe\left(Ti-Cr\right)}^{*},$ and $D_{Cr\left(Ti-Fe\right))}^{*}$ are shown in Table 5.

Comparing the average values in Table 5, the following relationships are obtained:

$$\frac{\bar{{\tilde{D}}_{FeFe}^{Ti}\left(1273\;K\right)}}{\bar{{\tilde{D}}_{CrCr}^{Ti}}\left(1273\;K\right)}=3.66$$

$$\frac{\bar{{\tilde{D}}_{FeFe}^{Ti}\left(1373\;K\right)}}{\bar{{\tilde{D}}_{CrCr}^{Ti}}\left(1373\;K\right)}=3.42$$

It is shown that the average interdiffusion coefficient and average impurity diffusion coefficient of Fe elements in bcc-Ti-Fe-Cr alloy are higher than those of Cr elements. Then, the average diffusion coefficients of the same element at different temperatures were compared to analyze the effect of increasing temperature on the diffusion behavior of Fe and Cr elements. Specifically, they are as follows:

$$\frac{\bar{{\tilde{D}}_{FeFe}^{Ti}}\left(1373\;K\right)}{\bar{{\tilde{D}}_{FeFe}^{Ti}}\left(1273\;K\right)}=3.65\;\;\;\;\;\;\;\;\;\;\;\;\;\;\;\;\;\;\;\;\frac{\bar{{\tilde{D}}_{CrCr}^{Ti}}\left(1373\;K\right)}{\bar{{\tilde{D}}_{CrCr}^{Ti}}\left(1273\;K\right)}=3.91$$

$$\frac{\bar{D_{Fe\left(Ti-Cr\right)}^{*}}\left(1373\;K\right)}{\bar{D_{Fe\left(Ti-Cr\right)}^{*}}\left(1273\;K\right)}=4.12\;\;\;\;\;\;\;\;\;\;\;\frac{\bar{D_{Cr\left(Ti-Fe\right)}^{*}}\left(1373\;K\right)}{\bar{D_{Cr\left(Ti-Fe\right)}^{*}}\left(1273\;K\right)}=3.73$$

The above equations show that the diffusion coefficient increases by about three times when the temperature increases by 100 K.

**Figure 5:**
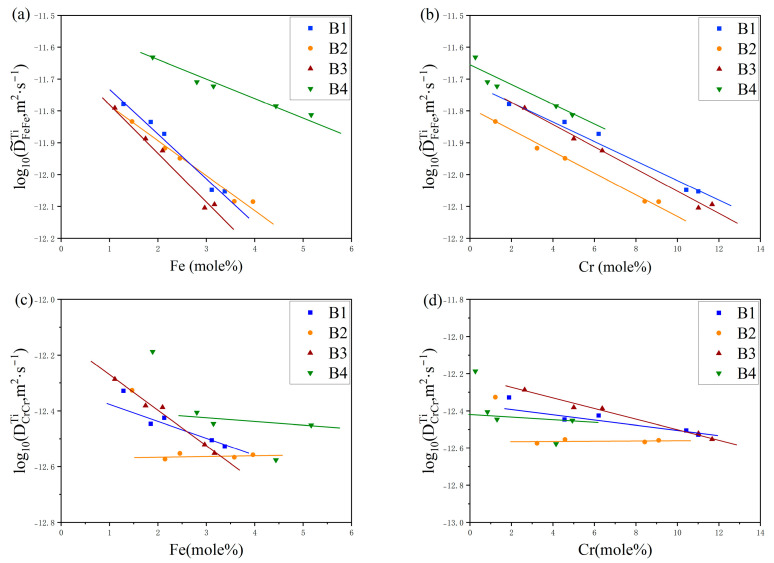
The variations in ternary inter-diffusion coefficients with the compositions: (**a**) ${\tilde{D}}_{FeFe}^{Ti}$ with Fe, (**b**) ${\tilde{D}}_{FeFe}^{Ti}$ with Cr, (**c**) ${\tilde{D}}_{CrCr}^{Ti}$ with Fe, and (**d**) ${\tilde{D}}_{CrCr}^{Ti}$ with Cr at 1273 K.

**Figure 6:**
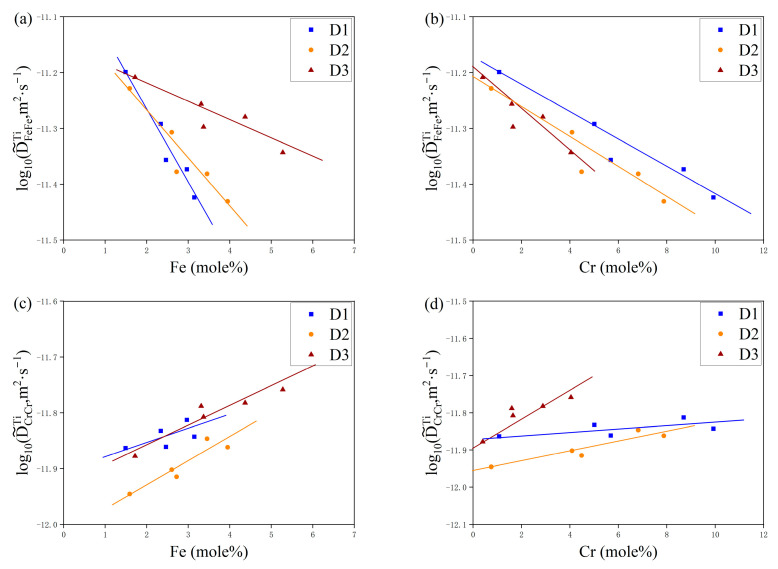
The variations in the ternary inter-diffusion coefficients with the compositions: (**a**) ${\tilde{D}}_{FeFe}^{Ti}$ with Fe, (**b**) ${\tilde{D}}_{FeFe}^{Ti}$ with Cr, (**c**) ${\tilde{D}}_{CrCr}^{Ti}$ with Fe, and (**d**) ${\tilde{D}}_{CrCr}^{Ti}$ with Cr at 1373 K.

In Figure 5 and Figure 6, the dependencies of the primary interdiffusion coefficients, namely ${\tilde{D}}_{FeFe}^{Ti}$ and ${\tilde{D}}_{CrCr}^{Ti}$, on the concentrations of Fe and Cr are depicted for temperatures of 1273 K and 1373 K, respectively. These figures illustrate how the diffusion coefficients adjust as a function of varying elemental compositions at the specified thermal conditions. The different colored markers in the figure represent different diffusion couples. It can be found in Figure 5 and Figure 6 that the main interdiffusion coefficient ${\tilde{D}}_{FeFe}^{Ti}$ decreases with the increase in Fe and Cr elements. However, the main interdiffusion coefficient, ${\tilde{D}}_{CrCr}^{Ti}$, exhibits a completely different phenomenon at 1000 °C and 1100 °C. As shown in Figure 5 and Figure 6, ${\tilde{D}}_{CrCr}^{Ti}$ slowly decreases with the increase in Fe and Cr elements at 1000 °C, but increases with the increase in Fe and Cr elements at 1100 °C.

## 5. Atomic Mobility Optimization

Employing Thermo-Calc (Educational 2023b) software, which is seamlessly integrated with the DICTRA module, facilitated the refinement of the atomic mobility parameters within the ternary bcc Ti-Fe-Cr alloy system. The results of this optimization process are systematically documented in Table 6, as found in the referenced literature. Based on the thermodynamic parameters of binary system and the ternary diffusion coefficient calculated at 1273 K and 1373 K, the ternary atomic mobility parameters were optimized.

**Figure 7:**
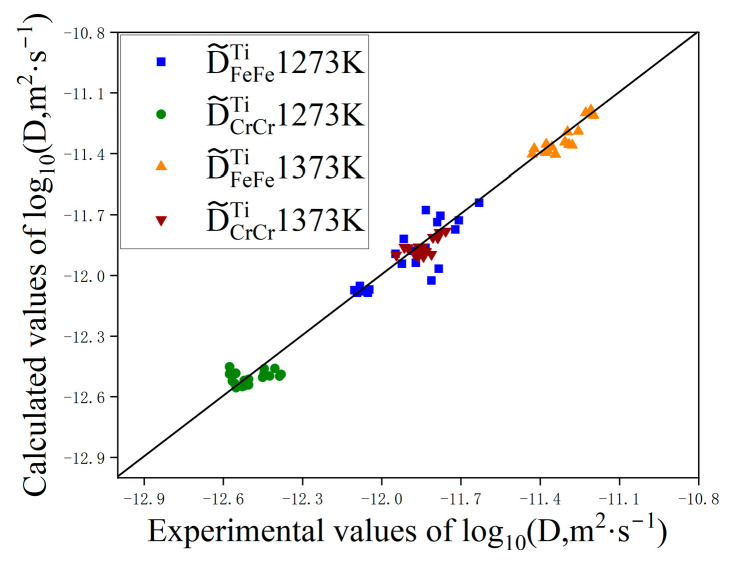
Analysis of main inter-diffusion coefficients in bcc Ti-Fe-Cr alloys, showcasing a comparison of logarithmically expressed calculated values against those obtained experimentally.

The refined atomic mobility parameters were then applied to calculate the ternary diffusion coefficients for the Ti-Fe-Cr system, ensuring a rigorous comparison with the experimental data at the aforementioned temperatures. A comparison between the calculated and experimental values of the diffusion coefficient is shown in Figure 7. All calculated diffusion coefficients are within acceptable deviations, so the data points are distributed near the x = y line. In the graph, it can be seen that the calculated values of the main diffusion coefficients ${\tilde{D}}_{FeFe}^{Ti}$ and ${\tilde{D}}_{CrCr}^{Ti}$ at 1373 K have very small errors compared to the measured values, indicating that the data points are closely aligned with the straight line. The distribution of data points at 1273 K is relatively discrete; except for a few points that deviate from the straight line, most points are distributed on both sides of the line. The above results indicate a strong consistency between the calculated diffusion coefficient values and the experimental measurement values, thus confirming the reliability and accuracy of the optimization results in Table 6.

**Figure 8:**
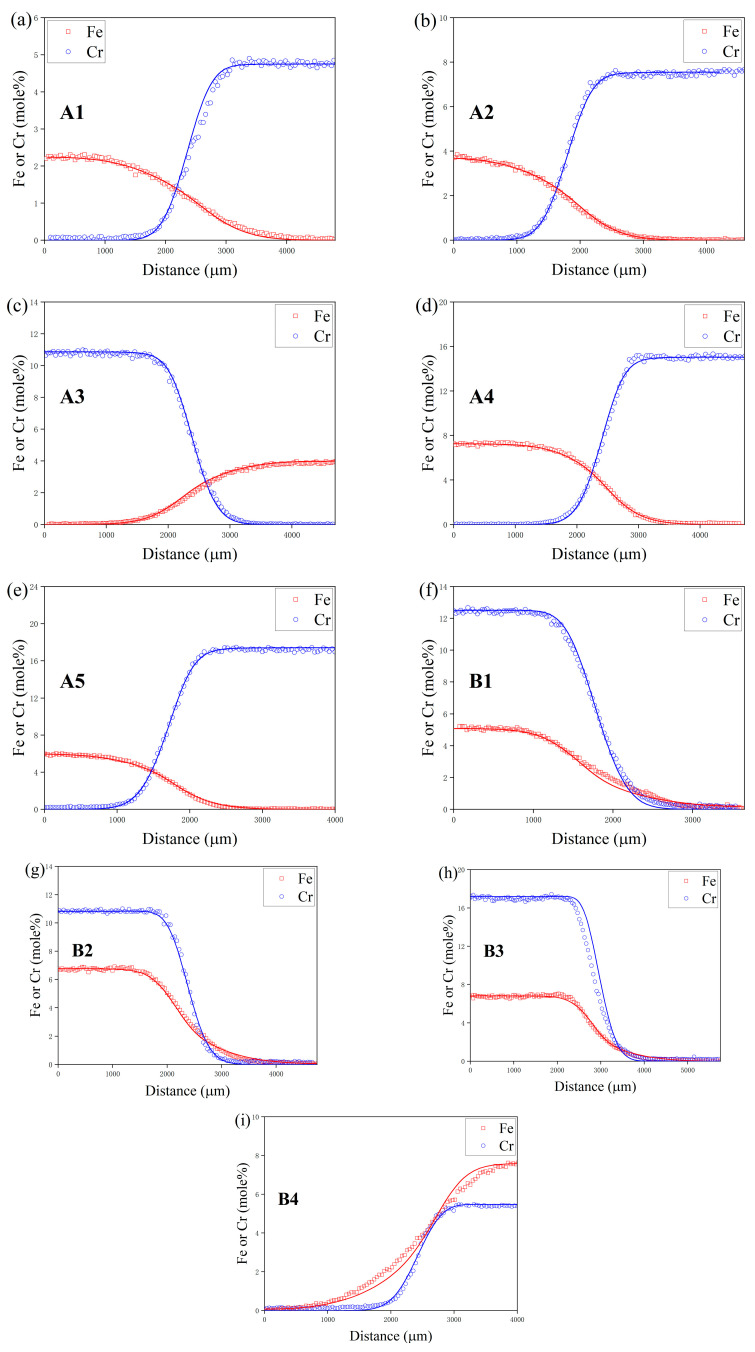
Comparison between the experimental and simulated composition-distance profiles of diffusion couple (**a**) A1 at 1273 K, (**b**) A2 at 1273 K, (**c**) A3 at 1273 K, (**d**) A4 at 1273 K, (**e**) A5 at 1273 K, (**f**) B1 at 1273 K, (**g**) B2 at 1273 K, (**h**) B3 at 1273 K and (**i**) B4 at 1273 K, where the simulated results is illustrated by the solid line.

**Figure 9:**
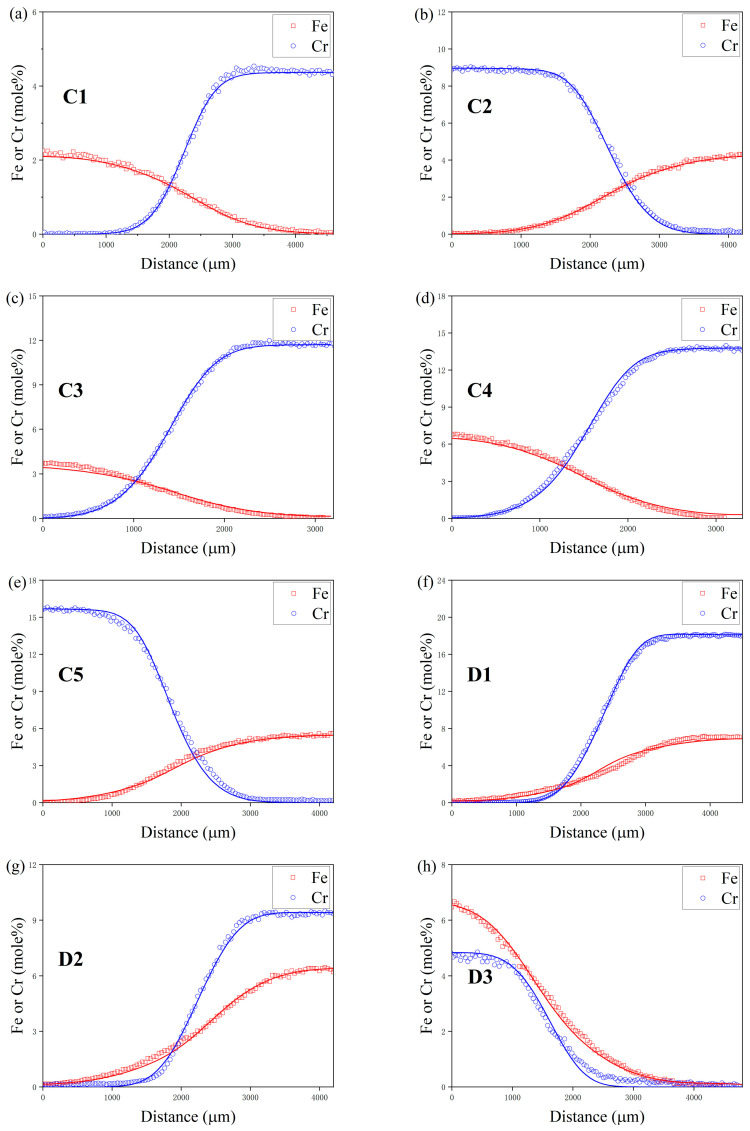
Comparison between the experimental and simulated composition-distance profiles of diffusion couple (**a**) C1 at 1373 K, (**b**) C2 at 1373 K, (**c**) C3 at 1373 K, (**d**) C4 at 1373 K, (**e**) C5 at 1373 K, (**f**) D1 at 1373 K, (**g**) D2 at 1373 K and (**h**) D3 at 1373 K. The solid line denotes the simulation result.

**Figure 10:**
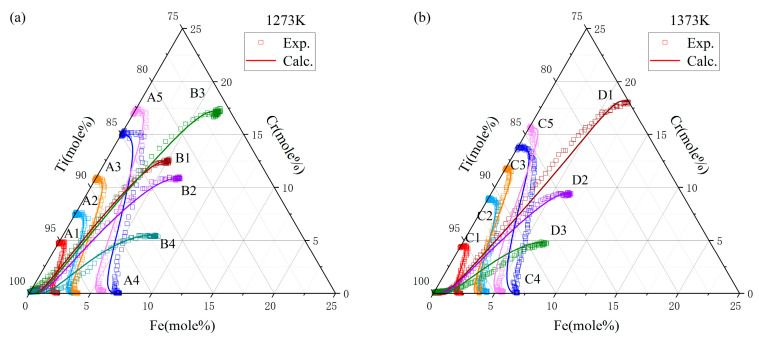
Discrepancy analysis between the modeled and observed diffusion channel values in the Ti-Fe-Cr system post-annealing at (**a**) 1273 K for 44 h and (**b**) 1373 K for 22 h.

A diffusion model was developed to mimic the atomic diffusion behaviors under identical initial conditions and thermal treatments. Figure 8 and Figure 9 depict the component distance curves for nine diffusion couple pairs annealed at 1273 K and eight pairs at 1373 K, as determined through experiments and simulations. The figures demonstrate that the simulation curves for the majority of the diffusion couples align closely with the experimental data. However, discrepancies are noted in some simulations, such as for diffusion couples D1 and D3, where the component gradient in the diffusion zone is underestimated, resulting in a simulated diffusion coefficient larger than the experimental findings. This discrepancy might stem from the differential diffusion rates of Fe and Cr, leading to fitting errors during the composition distance curve analysis via electron probe microscopy, as it may not accurately represent the true terminal positions of Fe and Cr diffusion. Figure 10 showcases the comparison of the simulated diffusion paths against the experimental paths for 17 diffusion couple pairs. The substantial concordance between the simulation outcomes and the experimental data underscores the high precision of the derived atomic mobility parameters.

## 6. Conclusions

In this investigation, the researchers fabricated two groups of bcc Ti-Fe-Cr diffusion couples, which underwent annealing processes at temperatures of 1273 K and 1373 K for durations of 44 h and 22 h, respectively. This study harnessed the methodologies devised by Whittle–Green and Hall to extract the ternary interdiffusion and impurity diffusion coefficients from the collected data. The atomic mobility parameters of the bcc-Ti-Fe-Cr system were optimized and evaluated using the CALPHAD method. The following conclusions were obtained:

(1) Observations from the study elucidate that Fe manifests a higher diffusion velocity than Cr in the bcc Ti-Fe-Cr alloy when subjected to the same thermal conditions. The diffusion rates of both elements in the alloy are positively correlated with the temperature; as the temperature increases, the diffusion rate also increases.
(2) At 1273 K and 1373 K, the main diffusion coefficient ${\tilde{D}}_{FeFe}^{Ti}$ decreases with the increase in Fe and Cr elements. The main diffusion coefficient ${\tilde{D}}_{CrCr}^{Ti}$ slowly decreases with the increase in Fe and Cr elements at 1273 K, but increases with the increase in Fe and Cr elements at 1373 K.
(3) Using the atomic mobility parameters, the diffusion process of alloy in diffusion couple was simulated in DICTRA software. A comparative analysis of the simulation outcomes with the experimental data highlighted minor discrepancies, all of which fell within the bounds of acceptable error margins. This congruence attests to the reliability and accuracy of the atomic mobility parameters, validating the comprehensive approach employed in matching the experimental findings with the theoretical models.

## Figures and Tables

**Table 1:** Terminal compositions of the diffusion couples.

Temperature (K)	Diffusion Couples	Composition (at.%)
1273	A1	Ti-2.20Fe/Ti-4.69Cr
	A2	Ti-3.72Fe/Ti-7.55Cr
	A3	Ti-4.00Fe/Ti-10.86Cr
	A4	Ti-7.25Fe/Ti-15.04Cr
	A5	Ti-6.00Fe/Ti-17.40Cr
	B1	Pure Ti/Ti-5.22Fe-12.25Cr
	B2	Pure Ti/Ti-6.77Fe-10.82Cr
	B3	Pure Ti/Ti-6.81Fe-17.19Cr
	B4	Pure Ti/Ti-7.59Fe-5.47Cr
1373	C1	Ti-2.15Fe/Ti-4.37Cr
	C2	Ti-4.29Fe/Ti-8.96Cr
	C3	Ti-3.73Fe/Ti-11.73Cr
	C4	Ti-6.81Fe/Ti-13.82Cr
	C5	Ti-5.50Fe/Ti-15.70Cr
	D1	Pure Ti/Ti-7.08Fe-18.06Cr
	D2	Pure Ti/Ti-6.44Fe-9.41Cr
	D3	Pure Ti/Ti-6.70Fe-4.83Cr

**Table 2:** Experimental interdiffusion coefficients in bcc Ti-Fe-Cr alloys at 1273 K.

Diffusion Couple	Composition (at.%)		Interdiffusion Coefficients			
	Fe	Cr	${\tilde{D}}_{FeFe}^{Ti}$ (×10^−12^ m^2^·s^−1^)	${\tilde{D}}_{FeCr}^{Ti}$ (×10^−14^ m^2^·s^−1^)	${\tilde{D}}_{CrFe}^{Ti}$ (×10^−13^ m^2^·s^−1^)	${\tilde{D}}_{CrCr}^{Ti}$ (×10^−13^ m^2^·s^−1^)
A1-B1	1.29	1.88	1.67 ± 0.03	−9.51 ± 0.35	−2.39 ± 0.84	4.70 ± 0.13
A1-B2	1.47	1.22	1.47 ± 0.06	−16.19 ± 1.02	−3.27 ± 1.27	4.72 ± 0.39
A1-B3	1.11	2.63	1.62 ± 0.02	−10.47 ± 0.45	1.73 ± 0.78	5.17 ± 0.09
A1-B4	1.89	0.26	2.33 ± 0.01	39.91 ± 4.63	1.00 ± 0.26	6.50 ± 0.40
A2-B1	1.85	4.55	1.46 ± 0.01	2.20 ± 0.02	1.13 ± 0.36	3.58 ± 0.08
A2-B2	2.15	3.23	1.21 ± 0.04	−3.48 ± 0.88	−2.84 ± 0.14	2.67 ± 0.01
A2-B3	1.74	5.00	1.30 ± 0.03	−1.79 ± 0.69	3.21 ± 0.11	4.16 ± 0.01
A2-B4	2.80	0.84	1.95 ± 0.04	23.87 ± 0.14	−0.37 ± 0.88	3.93 ± 0.14
A3-B1	2.13	6.20	1.34 ± 0.02	2.99 ± 0.02	−0.31 ± 0.38	3.76 ± 0.02
A3-B2	2.45	4.58	1.12 ± 0.07	−1.83 ± 0.50	−5.18 ± 0.64	2.80 ± 0.12
A3-B3	2.09	6.38	1.19 ± 0.03	−0.08 ± 0.62	1.33 ± 0.09	4.10 ± 0.02
A3-B4	3.15	1.30	1.90 ± 0.03	16.91 ± 0.09	−0.62 ± 0.89	3.58 ± 0.05
A4-B1	3.38	11.00	0.89 ± 0.01	3.28 ± 0.22	1.26 ± 0.11	2.97 ± 0.11
A4-B2	3.96	9.10	0.82 ± 0.09	0.31 ± 0.58	−0.87 ± 0.18	2.77 ± 0.10
A4-B3	3.17	11.67	0.81 ± 0.01	1.14 ± 0.06	1.52 ± 0.63	2.81 ± 0.06
A4-B4	5.17	4.93	1.54 ± 0.07	19.79 ± 0.84	−0.66 ± 0.47	3.54 ± 0.13
A5-B1	3.11	10.24	0.90 ± 0.02	5.00 ± 0.09	1.22 ± 0.18	3.13 ± 0.04
A5-B2	3.58	8.43	0.82 ± 0.04	1.83 ± 0.52	−0.79 ± 0.13	2.71 ± 0.02
A5-B3	2.96	11.01	0.79 ± 0.08	2.65 ± 0.19	1.10 ± 0.01	3.01 ± 0.05
A5-B4	4.44	4.15	1.64 ± 0.05	15.00 ± 0.52	−0.06 ± 0.62	2.65 ± 0.01

**Table 3:** Experimental interdiffusion coefficients in bcc Ti-Fe-Cr alloys at 1373 K.

Diffusion Couple	Composition (at.%)		Interdiffusion Coefficients			
	Fe	Cr	${\tilde{D}}_{FeFe}^{Ti}$ (×10^−12^ m^2^·s^−1^)	${\tilde{D}}_{FeCr}^{Ti}$ (×10^−13^ m^2^·s^−1^)	${\tilde{D}}_{CrFe}^{Ti}$ (×10^−13^ m^2^·s^−1^)	${\tilde{D}}_{CrCr}^{Ti}$ (×10^−12^ m^2^·s^−1^)
C1-D1	1.49	1.08	6.33 ± 0.02	2.70 ± 0.34	−0.49 ± 0.06	1.37 ± 0.05
C1-D2	1.59	0.75	5.91 ± 0.07	1.29 ± 0.53	−6.63 ± 1.27	1.13 ± 0.06
C1-D3	1.72	0.40	6.19 ± 0.04	2.55 ± 1.26	2.66 ± 0.97	1.33 ± 0.08
C2-D1	2.34	5.01	5.10 ± 0.11	2.98 ± 0.28	−2.92 ± 1.50	1.47 ± 0.02
C2-D2	2.60	4.09	4.93 ± 0.03	2.03 ± 0.43	−9.81 ± 0.03	1.25 ± 0.04
C2-D3	3.31	1.60	5.54 ± 0.03	3.09 ± 0.28	3.53 ± 1.93	1.63 ± 0.09
C3-D1	2.46	5.69	4.40 ± 0.06	3.87 ± 0.01	2.92 ± 0.50	1.38 ± 0.07
C3-D2	2.72	4.48	4.19 ± 0.08	3.81 ± 0.31	−7.57 ± 0.98	1.22 ± 0.06
C3-D3	3.37	1.65	5.04 ± 0.08	7.80 ± 0.06	4.25 ± 2.21	1.56 ± 0.05
C4-D1	3.15	9.93	3.77 ± 0.01	3.39 ± 0.35	−0.84 ± 0.18	1.44 ± 0.03
C4-D2	3.95	7.88	3.71 ± 0.07	2.41 ± 0.34	−9.36 ± 0.20	1.37 ± 0.04
C4-D3	5.28	4.04	4.54 ± 0.03	3.06 ± 0.59	−1.75 ± 1.34	1.74 ± 0.06
C5-D1	2.97	8.70	4.23 ± 0.01	3.02 ± 0.43	−6.67 ± 0.05	1.54 ± 0.01
C5-D2	3.45	6.82	4.16 ± 0.06	1.95 ± 0.23	−11.45 ± 1.14	1.42 ± 0.02
C5-D3	4.37	2.88	5.26 ± 0.01	1.77 ± 0.08	−1.36 ± 1.91	1.65 ± 0.04

**Table 4:** Experimental impurity diffusion coefficients in bcc Ti-Fe-Cr alloys.

Temperature (K)	Composition (at.%)	Impurity Diffusion Coefficients (×10^−12^ m^2^·s^−1^)	Composition (at.%)	Impurity Diffusion Coefficients (×10^−12^ m^2^·s^−1^)
1273	$D_{Fe\left(Ti-4.76Cr\right)}^{*}$	1.750 ± 0.40	$D_{Cr\left(Ti-2.24Fe\right)}^{*}$	0.640 ± 0.46
	$D_{Fe\left(Ti-7.57Cr\right)}^{*}$	1.256 ± 0.99	$D_{Cr\left(Ti-3.69Fe\right)}^{*}$	0.266 ± 0.13
	$D_{Fe\left(Ti-10.79Cr\right)}^{*}$	1.002 ± 0.27	$D_{Cr\left(Ti-3.88Fe\right)}^{*}$	0.383 ± 0.21
	$D_{Fe\left(Ti-15.06Cr\right)}^{*}$	0.560 ± 0.52	$D_{Cr\left(Ti-7.23Fe\right)}^{*}$	0.441 ± 0.23
	$D_{Fe\left(Ti-17.20Cr\right)}^{*}$	0.521 ± 0.22	$D_{Cr\left(Ti-5.92Fe\right)}^{*}$	0.227 ± 0.51
1373	$D_{Fe\left(Ti-4.41Cr\right)}^{*}$	5.363 ± 0.15	$D_{Cr\left(Ti-2.19Fe\right)}^{*}$	1.323 ± 0.45
	$D_{Fe\left(Ti-8.93Cr\right)}^{*}$	4.222 ± 0.87	$D_{Cr\left(Ti-4.30Fe\right)}^{*}$	1.458 ± 0.17
	$D_{Fe\left(Ti-11.75Cr\right)}^{*}$	3.162 ± 0.37	$D_{Cr\left(Ti-3.73Fe\right)}^{*}$	1.013 ± 0.38
	$D_{Fe\left(Ti-13.73Cr\right)}^{*}$	3.355 ± 0.44	$D_{Cr\left(Ti-6.81Fe\right)}^{*}$	1.844 ± 0.61
	$D_{Fe\left(Ti-15.70Cr\right)}^{*}$	4.916 ± 0.25	$D_{Cr\left(Ti-5.52Fe\right)}^{*}$	1.678 ± 0.33

**Table 5:** Statistical results of diffusion coefficients obtained experimentally.

Temperature (K)	Values	Diffusion Coefficients (m^2^·s^−1^)			
		${\tilde{D}}_{FeFe}^{Ti}$	${\tilde{D}}_{CrCr}^{Ti}$	$D_{Fe\left(Ti-Cr\right)}^{*}$	$D_{Cr\left(Ti-Fe\right)}^{*}$
1273	Minimum	7.86 × 10^−13^	2.65 × 10^−13^	5.21 × 10^−13^	2.27 × 10^−13^
	Maximum	2.33 × 10^−12^	6.50 × 10^−13^	1.75 × 10^−12^	6.40 × 10^−13^
	Average	1.34 × 10^−12^	3.66 × 10^−13^	1.02 × 10^−12^	3.91 × 10^−13^
1373	Minimum	2.71 × 10^−12^	1.13 × 10^−12^	3.16 × 10^−12^	1.01 × 10^−12^
	Maximum	6.33 × 10^−12^	1.74 × 10^−12^	5.36 × 10^−12^	1.84 × 10^−12^
	Average	4.89 × 10^−12^	1.43 × 10^−12^	4.20 × 10^−12^	1.46 × 10^−12^

**Table 6:** Atomic mobility parameters for the bcc phase of the Ti-Fe-Cr system.

Mobility	Parameters, J/mole	Reference
Mobilities of Ti		
$Q_{Ti}^{Ti}$	RTln(5.91 × 10^−5^exp(−23,700/RT) + 1.47 × 10^−8^ exp(−121,000/RT))	[52]
$Q_{Ti}^{Fe}$	−293,200 + RTln(0.21)	[53]
$Q_{Ti}^{Cr}$	−401,358−35.5 × T	[41]
Q 0TiTi,Fe	−208,542.06 + 185.09 × T	[53]
Q 0TiTi,Cr	221,687.185	[13]
Mobilities of Fe		
$Q_{Fe}^{Ti}$	RTln(7.8 × 10^−7^exp(−132,000/RT) + 2.7 × 10^−4^ exp(−230,300/RT))	[53]
$Q_{Fe}^{Fe}$	−258,194 + RTln(6.6 × 10^−4^)	[54]
$Q_{Fe}^{Cr}$	−332,000 + RTln(4.7 × 10^−5^)	[54]
Q 0FeTi,Fe	−692,842.42 + 480.24 × T	[53]
Q 0FeTi,Cr	−1,903,266.41 + 1759.71 × T	This work
Q 1FeTi,Cr	−1,671,434.55 + 1590.81 × T	This work
Q 0FeFe,Cr	256,342.886	[55]
Q 1FeFe,Cr	−407,725.609	[55]
Mobilities of Cr		
$Q_{Cr}^{Ti}$	RTln(1.6 × 10^−3^exp(−276,284/RT) + 8.6 × 10^−7^ exp(−156,991/RT))	[13]
$Q_{Cr}^{Fe}$	−266,300 + RTln(2.2 × 10^−2^)	[54]
$Q_{Cr}^{Cr}$	−441,974−16.94 × T	[41]
Q 0CrTi,Cr	163,816.65	[13]
Q 0CrTi,Fe	832,547.16−935.52 × T	This work
Q 1CrTi,Fe	2,693,787.95−2446.7 × T	This work
Q 0CrFe,Cr	273,329.671	[55]
Q 1CrFe,Cr	−19,802.888	[55]

## Data Availability

Data are contained within the article.
